# HOX cluster and their cofactors showed an altered expression pattern in eutopic and ectopic endometriosis tissues

**DOI:** 10.1186/s12958-021-00816-y

**Published:** 2021-09-01

**Authors:** Fereshteh Esfandiari, Fereshteh Chitsazian, Masoumeh Golestan Jahromi, Raha Favaedi, Masood Bazrgar, Reza Aflatoonian, Parvaneh Afsharian, Abbas Aflatoonian, Maryam Shahhoseini

**Affiliations:** 1 Department of Stem Cells and Developmental Biology, Cell Science Research Center, Royan Institute for Stem Cell Biology and Technology, ACECR, Tehran, Iran; 2 Department of Genetics, Reproductive Biomedicine Research Center, Royan Institute for Reproductive Biomedicine, ACECR, Hafez St.Resalat Ave, P.O. Box, 19395-4644 Banihashem St.Tehran, No. 2 Iran; 3 Research and Clinical Center for Infertility, Yazd Reproductive Sciences Institute, Shahid Sadoughi University of Medical Sciences, Bouali Ave; Safaeyeh, Yazd, Iran; 4 Department of Endocrinology and Female Infertility, Reproductive Biomedicine Research Center, Royan Institute for Reproductive Biomedicine, ACECR, Tehran, Iran; 5 Reproductive Epidemiology Research Center, Royan Institute for Reproductive Biomedicine, ACECR, Tehran, Iran; 6 Department of Cell and Molecular Biology, School of Biology, College of Science, University of Tehran, Tehran, Iran

**Keywords:** Endometriosis, Gene expression, HOX

## Abstract

Endometriosis is major gynecological disease that affects over 10% of women worldwide and 30%-50% of these women have pelvic pain, abnormal uterine bleeding and infertility. The cause of endometriosis is unknown and there is no definite cure mainly because of our limited knowledge about its pathophysiology at the cellular and molecular levels. Therefore, demystifying the molecular mechanisms that underlie endometriosis is essential to develop advanced therapies for this disease. In this regard, *HOX* genes are remarkable because of their critical role in endometrial development and receptivity during implantation, which is attributed to their ability to mediate some of the sex steroid functions during the reproductive period. Access to the expression profiles of these genes would provide the necessary information to uncover new genes for endometriosis and assist with disease diagnosis and treatment. In this study we demonstrate an altered expression pattern for the *HOX* clusters (A-D) and their cofactors in both eutopic and ectopic conditions compared to control tissue biopsies. Remarkably, most of the intensive changes occurred in eutopic samples from endometriosis patients compared to control tissue biopsies. Pathway analysis revealed the involvement of differentially expressed genes in cancer that correlate with an association between endometriosis and cancer. Our results suggest critical roles for the *HOX* cluster and their cofactors in endometriosis pathophysiology.

**Supplementary Information:**

The online version contains supplementary material available at 10.1186/s12958-021-00816-y.

## Background

Endometriosis is an estrogen-dependent gynecological disorder that affects 10%-15% of reproductive age women [1, 2]. It is characterized by the presence of endometrial tissues located outside of the uterus. The symptoms include dysmenorrhea, pelvic pain, pelvic masses, and infertility [3–6]. Social relationships, sexuality, and mental health are affected by endometriosis [7]. There is no definitive cure for all patients who suffer from endometriosis. Therefore, developing advanced efficient therapies in addition to prevention of endometriosis development and recurrence are of utmost importance [8].

It is essential to demystify the molecular mechanisms that underlie endometriosis in order to develop advanced therapies for this disease. In this regard, *HOX* genes are remarkable because they regulate endometrial development and its receptivity during implantation [9]. These genes are essential for endometrial growth, differentiation, and receptivity because they mediate some functions of the sex steroids during each reproductive period [10]. Dysregulation in *HOXD3* [10], *HOXA11* [11]*,* and *HOXB3* [10] have been reported in endometriosis patients. However, the changes in the expression levels of other *HOX* genes or *HOX*-cofactor genes in patients with endometriosis have not been elucidated and interactions between these genes remain to be determined.

Access to expression profiles of these genes would provide the information needed to uncover new genes that could assist with endometriosis diagnosis and treatment. In this study, we report about the expression profiles of *HOX* genes, their cofactors, and the interactions between these genes.

## Materials and methods

### Patients and tissue collection

The Ethics Committee of the Clinical and Research Centers for Infertility, Yazd, Iran and the Research Ethics Committee of Royan Institute, Tehran, Iran (IR.ACECR.ROYAN.REC.1396.238) approved this cross-sectional study. All methods were carried out in accordance with the approved guidelines [12]. Participants signed an informed consent in accordance with the guidelines of the Declaration of Helsinki (2000 revision) and tissue samples were collected after receipt of participants’ consent.

Study participants comprised 15 women with stages III or IV endometriosis who underwent laparoscopic surgery. The participants ranged in age from 24 to 38 years. Ectopic endometrial samples were collected from ectopic sites in the abdomen and eutopic endometrium samples were obtained by pipelle sampling. None of patients had evidence of any endometrial hyperplasia, visible endometrial hyperplasia or neoplasia, or any inflammatory diseases.

The control group consisted of 15 patients (22 to 36 years old) who underwent diagnostic laparoscopy for secondary infertility. Control group participants had no evidence of endometriosis, polyps, myoma, or reproductive inflammatory diseases and had at least one child conceived by natural pregnancy. Normal endometrial samples were taken via pipelle sampling from these women during diagnostic laparoscopy procedures. Confounding factors for samples are listed in Table S1.

All study participants did not have hormone therapy or an intrauterine device for three months before the surgical procedures, and all were in the follicular phase of their menstrual cycles. The obtained endometrial samples were placed immediately in an RNA protection reagent, RNAlater (Ambion, Austin, TX), frozen in liquid nitrogen, and stored at -80 °C.

### RNA extraction and cDNA synthesis

We pooled five tissue samples in the ectopic, eutopic, and normal groups as three biological repeats (each combined five tissue samples served as one biological repeat) and the following analyses were performed on the nine samples.

RNA extraction of collected tissues was performed by using an RNeasy Microarray Tissue Mini Kit (Qiagen, cat. no: 73304) according to the manufacturer’s instructions. RNA quality and concentration were measured by using a Nanodrop 2000 spectrophotometer (Thermo Scientific). Then, cDNA synthesis was performed with an RT2 First Strand Kit (Qiagen, cat. no: 330404).

### PCR array

The PCR array was conducted for the *HOX* family genes by using an RT2 Profiler PCR Array Human Homeobox Gene Kit (Qiagen, cat. no: PAHS-083Z) and an RT2 SYBR Green ROX qPCR Master Mix (Qiagen, cat. no: 330502) [13]. There were primer sets for 84 tests and the housekeeping genes on mentioned HOX PCR array kit, according to the manufacturer’s instructions. Cycling conditions for the StepOnePlus Real-time PCR system [14] were: an initial denaturation at 95 °C for 10 min followed by 40 cycles at 95 °C for 15 s, and 60 °C for one minute. Results were expressed as values of the cycle threshold [15] and then normalized to *GAPDH,* as the housekeeping gene (control gene). The 2^−∆∆Ct^ method was used to calculate fold changes in mRNA abundance.

### Statistical analysis

The statistical significance of difference in expression levels between groups was analyzed using the ANOVA test. *P*-values < 0.05 were considered to be statistically significant. Principal component analysis (PCA) [16] was carried out using Minitab 16 statistical software to simplify the large amount of data. PCA was applied on log2 of differentially expressed genes (DEGs) and non-DEGs data separately, to underlying cluster structures of endometrial samples.

Hierarchical clustering [17] was implemented by using the correlation coefficient and complete linkage in Minitab 16 statistical software [18]. The methods were performed twice with normalized gene expression data from the DEGs and non-DEGs among the sample groups, in three clusters.

### Construction of the gene co-expression network

The gene co-expression networks were constructed based on the normalized gene expression data. MATLAB was used to compute the Pearson correlation between each pair of genes, then the significant correlation pairs (*p* < 0.05) were imported into Cytoscape software (version 3.6.1) [19] for visualization and the circle algorithms were used [20]. The co-expression networks of the normal tissues were constructed.

### Coding gene functional analysis

Gene function was annotated based on Gene Ontology (GO), the intervention in other diseases, and expression in other tissues by using DAVID (https://david.ncifcrf.gov) [21] to clarify the function of the DEGs and the mechanism of endometriosis.

## Results

### Differentially expressed genes (DEGs)

**Fig. 1:**
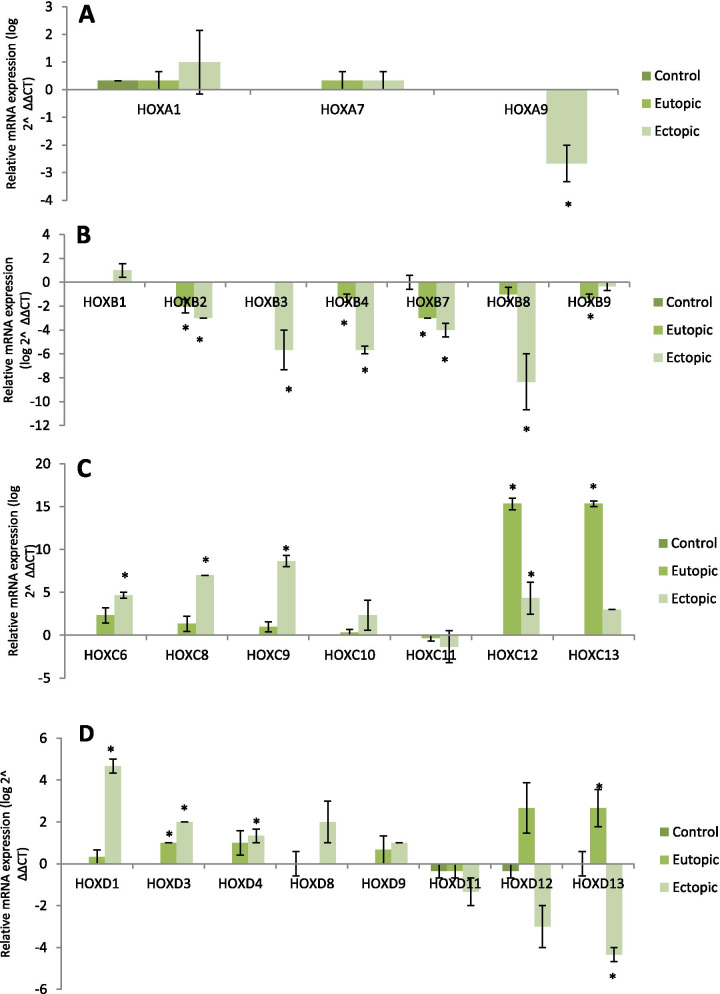
Gene expression array analysis for *HOX* genes. There were three biological repeats in each group. A, B, C and D in each graph show the nine data that were analyzed with SPSS software. *P* < 0.05 was considered to be significant

**Fig. 2:**
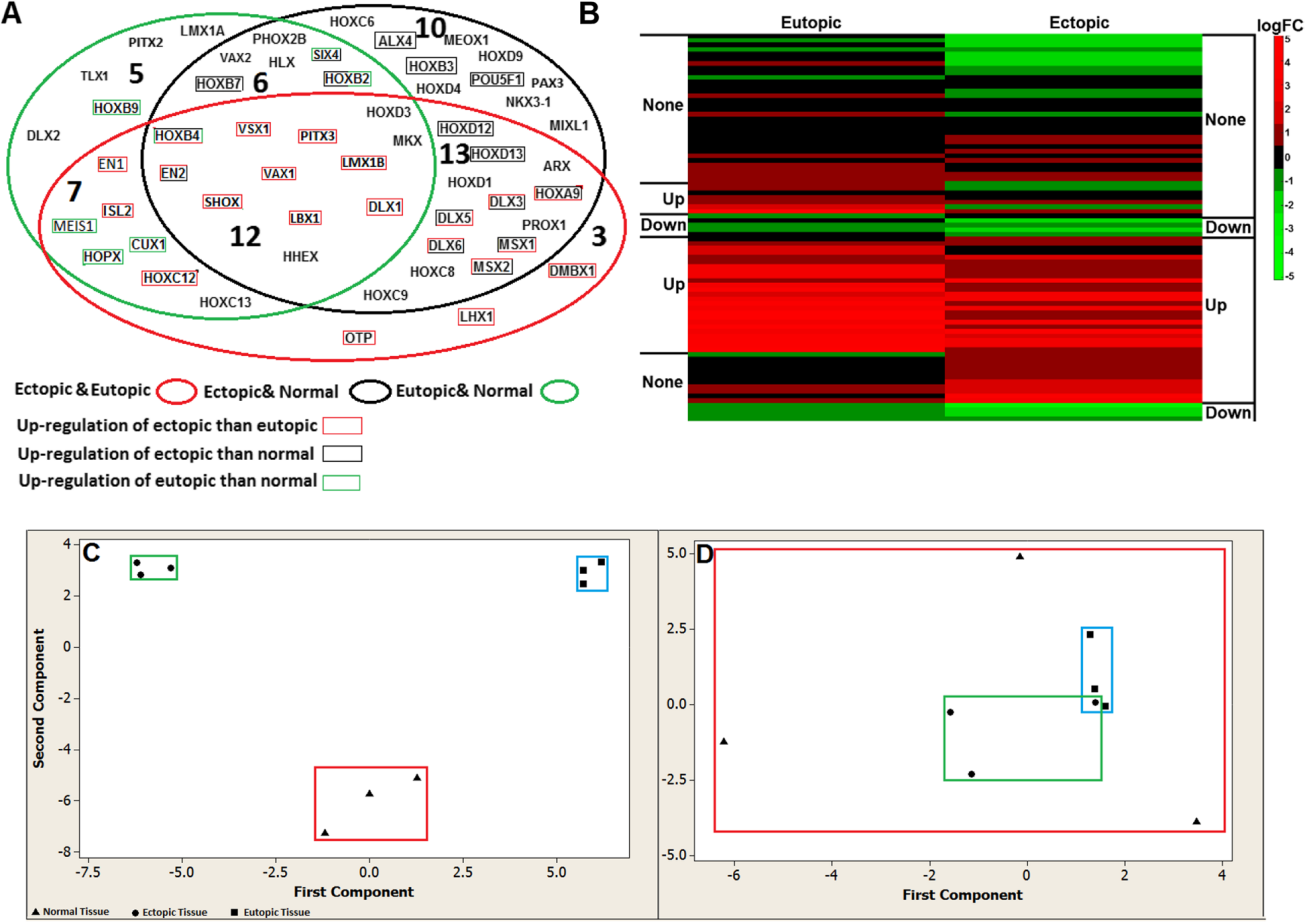
**A** Venn diagram for DEGs (*P* < 0.05). DEGs in the ectopic samples compared to normal samples (black ellipse), eutopic samples in comparison to normal samples (green ellipse), and ectopic samples compared to eutopic samples (red ellipse). Genes that were significantly upregulated are enclosed in black, green and red rectangles according to the above mentioned legend. In the multi-colored rectangles, each color represents upregulation in the relevant analysis in the corresponding agreement. **B** Heat map for all genes. Right column is logFC of ectopic samples compared to normal samples and the left column is the logFC of eutopic samples in comparison to normal samples. logFC: Logarithm fold change; UP: Significant upregulation; Down: Significant downregulation; None: Non-significant changes. **C**,**D** PCA analysis of gene expression in the nine sample groups. Triangle, circle, and square represent normal endometrial tissues of the control women, and ectopic endometrial tissues and eutopic endometrial tissues from patients, respectively. C: PCA of DEGs. D: PCA of non-DEGs

We evaluated 84 genes from the *HOX* clusters A-D and their cofactors in the control, eutopic, and ectopic tissue biopsies. Figure 1A-D and supplementary figures S1-S3 show the changes in expression patterns of *HOX* clusters A-D and their cofactors, respectively. Table S2 lists the genes that showed significant differential expression (DEGs) (*P* < 0.05) in the ectopic and eutopic groups compared to the control group in addition to DEGs in the ectopic compared to the eutopic group. Among the genes we evaluated in this study, 38.1% (No: 32/84) were non-DEG and about 61.9% (52/84) were differentially expressed between the ectopic, eutopic, and control groups. There were 18 genes from the DEG that were common among the studied groups (eutopic, ectopic and control tissues) (Table S2, Fig. 2 A,B). Table S2 lists the various cluster of genes that had different expression patterns in the studied groups.

For further investigation, we performed PCA on the nine studied samples from the ectopic, eutopic, and control groups by separate analysis of the DEGs and non-DEGs (Fig. 2 C,D). Each point in a PCA graph represented one sample and all samples were properly classified into separate clusters based on the DEGs; however, the non-DEGs could not correctly classify the same samples. Then, we performed hierarchical clustering in the DEGs (Fig. S4-A) and non-DEGs (Fig. S4-B) in the eutopic, ectopic, and control samples**.** Hierarchical clustering analysis for the DEGs showed that control tissues were clustered together, and the eutopic and ectopic samples were classified in separate clusters (Supplementary figure S4).

### Co-expression networks

**Fig. 3:**
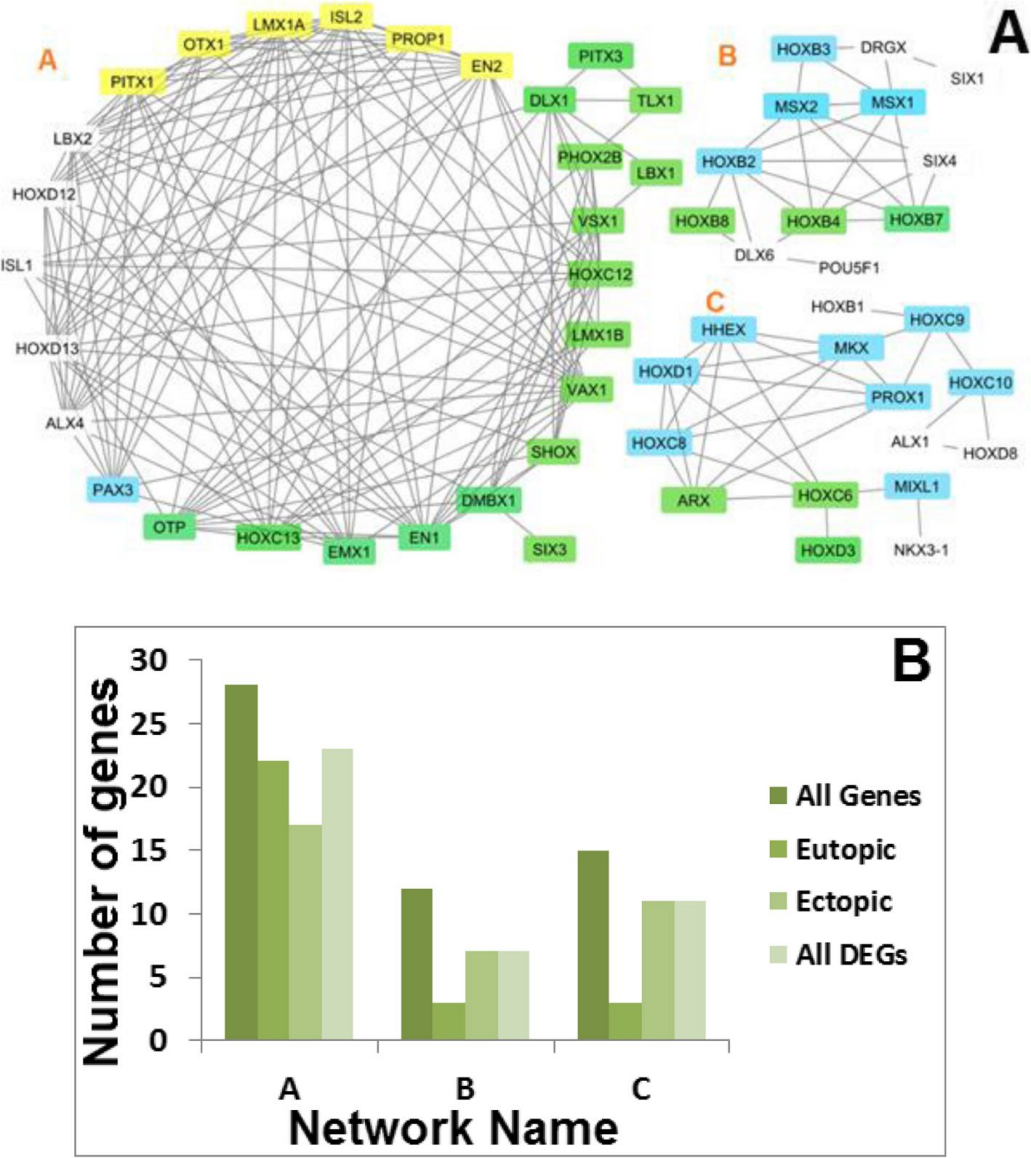
**A** Co-expression gene network. Blue, yellow, and green colors represent genes whose expressions significantly changed in the ectopic, eutopic and both tissues, respectively. **B** The number of genes with significant different expressions between sample tissues. All genes: The total number of nodes, Eutopic: Number of nodes with significant changes in gene expressions of the eutopic to control tissues, Ectopic: Number of nodes with significant changes in gene expression of the ectopic to control tissues, All DEGs: Number of nodes with significant changes in gene expressions of the patient to control tissues

We identified three co-expression networks based on pairwise correlation of the gene expression data and visualized them using Cytoscape (Fig. 3A). Network A was mainly occupied by genes that had significant expression changes in eutopic tissue compared to control tissue (78.6%); however, most genes in networks B and C showed significant expression changes in ectopic tissues. A large number of genes from all of the networks had significant expression changes in both ectopic and eutopic tissues compared to control tissues (Fig. 3B).

### Pathway analysis

The KEGG and Reactome databases were searched to determine the signaling pathways with involvement of networks A, B, and C. The number of genes grouped in networks A, B, and C were involved in “activation of anterior HOX genes in hindbrain development during early embryogenesis”. Also, the number of genes grouped in network A were involved in “signaling pathways regulating pluripotency of stem cells”. Some genes from network B were involved in “transcriptional misregulation in cancer” (Table 1).

**Table 1:** Pathways with genes in the constructed networks in the study

Gene symbol	*ISL1, OTX1*	*HOXB2, HOXB3, HOXB4*	*HOXB1, HOXD1, HOXD3*	*SIX1, SIX4*
Network name	A	B	C	B
Pathway name	S	R	R	T

R: Activation of anterior HOX genes in hindbrain development during early embryogenesis

S: Signaling pathways regulating pluripotency of stem cells

T: Transcriptional misregulation in cancer

### Functional analysis

DEGs and the genes in networks A, B, and C were mapped to the DAVID database to investigate GO, interference with other diseases, and expressions in other tissues (Tables 2, 3, S2 and S4).

**Table 2:** List of diseases that show interference by the DEGs and genes from the five networks

Description	Count	Percent	*P*-value
**DEGs in ectopic to control**			
Developmental	19	44.19	2.14E-11
Metabolic	17	39.53	0.038479
Neurological	12	27.91	0.024028
Cleft lip/Cleft palate	8	18.60	1.21E-05
Bone mineral density	7	16.28	1.64E-04
Vision	7	16.28	0.002492
Parkinson's disease	4	9.30	0.003099
Colobomatous microphthalmia	3	6.98	1.86E-04
Clubfoot	3	6.98	0.001869
Obstructive sleep apnea	3	6.98	0.00642
Hirschsprung's disease	2	4.65	0.034825
**DEGs in eutopic to control**			
Developmental	16	43.24	8.90E-09
Neurological	10	27.03	0.084075
Psychological	9	24.32	0.027439
Cleft lip/Cleft palate	6	16.22	8.20E-04
Parkinson's disease	5	13.51	1.46E-04
Autism	5	13.51	0.004516
Bone mineral density	5	13.51	0.007412
Vision	5	13.51	0.041046
**Genes in network A**			
Metabolic	13	46.43	0.03939
Developmental	12	42.86	1.51E-06
Psychological	11	39.29	1.60E-04
Autism	6	21.43	1.30E-04
Cleft lip/Cleft palate	6	21.43	2.09E-04
Bone mineral density	6	21.43	2.51E-04
Parkinson's disease	5	17.86	4.70E-05
Talipes equinovarus	2	7.14	0.005847
Neurodevelopmental psychiatric disorders	2	7.14	0.007304
SIDS/sudden infant death syndrome	2	7.14	0.038844
**Genes in network B**			
Cleft lip/Cleft palate	3	25	0.012792
Cleft lip with and without cleft palate	2	16.67	0.013342
Bone mineral density	3	25	0.013805
Developmental	4	33.33	0.023658
Sleep apnea, obstructive	2	16.67	0.026536
**Genes in network C**			
Developmental	7	46.67	1.47E-04
Clubfoot	3	20	1.97E-04
Bone mineral density	4	26.67	0.002301
Metabolic	9	60	0.004216

**Table 3:** Other tissues in which the DEGs and the genes in five networks are expressed

Description	Count		Percent		P-value
**DEGs in ectopic to control**					
Cerebellum	30		69.77	0.007552	
Salivary gland	30		69.77	0.023191	
Lymphoma Burkitts Raji	28		65.12	0.043332	
Adrenal cortex	13		30.23	0.004518	
Craniofacial	4		9.3	1.32E-06	
Retina	4		9.3	0.036957	
**DEGs in eutopic to control**					
Salivary gland		26	70.27	0.005005	
Lymphoma Burkitts Raji		25	67.57	0.005565	
Cerebellum		25	67.57	0.008935	
BM-CD33 + myeloid		24	64.86	0.010012	
Pons		17	45.95	0.028449	
Adrenal cortex		10	27.03	0.023199	
Craniofacial		4	10.81	7.19E-07	
Ovary neoplasia		3	8.11	0.033658	
**Genes in network A**					
Cerebellum		19	67.86	0.002102	
Salivary gland		19	67.86	0.005265	
Lymphoma Burkitts Raji		18	64.29	0.012292	
Prostate		17	60.71	0.015867	
BM-CD33 + myeloid		17	60.71	0.03038	
Pons		14	50	0.007547	
Eye_normal		13	46.43	0.032975	
Craniofacial		2	7.14	0.012725	
**Genes in network B**					
Uterine tumor_disease	7		58.33	0.003695	
Embryo_development	8		66.67	0.01666	
Cingulate cortex	5		41.67	0.025441	
Heart	6		50	0.047369	
**Genes in network C**					
Subthalamicnucleus	6		40	0.020087	
Skin_normal	7		46.67	0.027182	
Embryo_development	10		66.67	0.032412	
Germ cell tumor_disease	8		53.33	0.038866	

#### Analysis of interference with other diseases

Many DEGs in ectopic tissues play a role in developmental, neurological, and metabolic diseases. Numerous DEGs in eutopic tissues are also involved in developmental and neurological disease classes such as Cleft lip, Cleft palate, and Parkinson's disease. As shown in Table 2, the genes in networks A, B, and C were mostly involved in developmental diseases, while the genes in network A were involved in metabolic diseases.

#### Expressions in other tissues

Many DEGs in the eutopic and ectopic tissues were also expressed in different tissues, such as the cerebellar tissue, craniofacial tissue, salivary glands, and the adrenal cortex. Moreover, DEGs in the eutopic tissues were also identified in Burkitt lymphoma Raji and BM-CD33 + Myeloid (Table 3).

Network A genes were expressed in the cerebellar, craniofacial, salivary gland, lymphoma Burkitts Raji and BM-CD33 + Myeloid tissues. Genes in network B were expressed in uterine tumor tissue and embryo development. Network C genes were identified in embryo development (Table 3).

#### Gene Ontology (GO) annotation analysis

On the basis of the GO annotation, most DEGs in eutopic and ectopic tissues were involved in the transcription and development or morphogenesis of various systems. “Anterior/posterior pattern specification”, “embryonic skeletal system morphogenesis”, “dopaminergic neuron differentiation”, and “multicellular organism development” were some of the most important pathways listed for DEGs in ectopic tissues. Eutopic tissue DEGs were also involved in the “multicellular organism development”, “neuron development”, and “dopaminergic neuron differentiation” pathways (Table S3, S4).

“Anterior/posterior pattern specification”, multicellular organism development and “skeletal system morphogenesis” were listed for all of the networks (A, B, and C). Nervous system-related pathways like dopaminergic neuron differentiation and neuron development were listed for networks A and B (Tables S3, S4).

## Discussion

In this study, we have observed disruptions in expression patterns of *HOX* genes and their cofactors in eutopic and ectopic samples from endometriosis patients compared to samples derived from women without endometriosis.

The data demonstrated that most of the evaluated genes had altered expression patterns in the eutopic and ectopic samples from endometriosis patients compared to the control samples. Genes involved in the *HOXA-D* clusters showed disturbed patterns of expression in tissues derived from women with endometriosis compared to the control tissues. In line with our findings, several studies have reported an association between *HOX* genes and endometriosis, as well as cancer [10, 22, 23]. Differential expressions of *HOXB2, B3* and *B4* [10, 24], and the *HOXD2* and *D3* genes [10], which we detected in the current study, agreed with previous reports on endometriosis. Altered expressions of *HOXB2, B3, B4, B7,* and *B9,* which we observed in the endometriosis samples, correlated with previous reports for these genes in endometriosis and in progression and development of many cancers [24–26]. Here we reported misregulation of *HOXC* cluster genes in endometriosis. Their involvement with endometriosis, cancer, and the female reproductive tract have been shown previously [10, 27, 28]. Moreover, we reported contrariwise expression patterns for *HOXD12* and *HOXD13* (downregulated) and *HOXD1* and *D3* (upregulated) in this study on endometriotic lesions. Upregulated *HOXD* genes have a correlation with their expression profiles in some carcinomas [29].

*HOXC12*, *EN1*, *SHOX*, *TLX1*, *VAX1*, *VSX1*, *PAX3*, *PITX3*, *DLX1,* and *LBX1* showed intensive changes as they are upregulated in eutopic tissues of women with endometriosis compared to the control group. Accordingly, we reported that most of these genes were hypomethylated, which correlated with elevated expressions of these genes in endometriosis patients [30]. The expression level of *HOXC12* is increased in breast tumor samples [31] and *TLX1* shows misregulation in cancer [32]. Therefore, our finding re-enforces the previously reported association between cancer and endometriosis [33, 34]. There is *PAX3* expression in cytotrophoblast cells and decidua cells during early pregnancy [35], so its disturbed expression might be involved in implantation defects in endometriosis. *EN1*, *PITX3,* and *SHOX2* are involved in neuron formation in eutopic and ectopic tissues [36, 37], and the altered expressions in these genes may be related to nerve formation and pain sensation in endometriosis patients [38].

Most DEG genes in network A showed significantly altered expressions in ectopic tissues. These genes are mainly involved in development and nerve function. In contrast, most genes categorized in networks B and C showed significantly altered expressions in eutopic tissue and are involved in developmental functions (Fig. 3A).

Our analysis indicated that ectopic and eutopic DEG genes are mainly expressed in the salivary gland and Burkett's lymphoma (Table 3). We detected pathways related to stem cell pluripotency for DEGs, which was in line with previous reports for this pathway in endometriosis [39, 40]. Moreover, we identified a “transcriptional misregulation in cancer” pathway for the studied genes that correlated with a previously proven relationship between endometriosis and cancer [41, 42].

*HOX* gene expressions are regulated by the hormones estradiol and progesterone. Endocrine regulation of *HOX* gene expressions is important for reproduction [27, 43]. Because endometriosis is associated with disturbed hormonal function (estrogen hyperactivity and progesterone resistance) [44], it is possible that an altered expression profile of *HOX* in endometriosis tissues is a result of hormonal dysregulation.

The results of this study provide preliminary evidence for the involvement of certain pathways (neural and immune system, as well as cancer) in endometriosis because the DEG genes identified by our study are reported to play a role in the above pathways. However, the role of these pathways in endometriosis should be investigated. It remains to be determined how neurogenesis or cancer pathways induce endometriosis or promote the development of this disease.

Recently developed endometriosis organoids are suitable pre-clinical models to investigate molecular mechanisms that underlie this disease [30, 45]. We suggest knock out the upregulated genes with extensive fold changes like *HOXC12* in endometriosis organoids and over-express these genes in endometrial organoids developed from non-endometriosis patients to explore the role of these genes in the development of endometriosis. Endometriosis/endometrium organoids and stromal cell cultures provide a suitable platform to investigate the role of progesterone and estrogen in the disturbed expression profiles of *HOX* genes.

## Conclusion

Our study provides the first insight into the expression pattern of *HOX* clusters and their cofactors in endometriosis. The results suggest an important role for these genes in the pathophysiology of this disease. Some genes from this platform should be additionally investigated in the future because of their thousand-fold changes in endometriosis tissue biopsies compared to control tissue biopsies.

## Supplementary Information

**Additional file 1.**

## Data Availability

Data sharing is not applicable to this article as no datasets were generated or analyzed during the current study.

## Abbreviations

cDNA: Complementary DNA
DAVID: Database for Annotation, Visualization, and Integrated Discovery
DEG: Differentially expressed gene
GO: Gene Ontology
KEGG: Kyoto Encyclopedia of Genes and Genomes
non-DEG: Non-differentially expressed gene
PCA: Principal component analysis
CtoN: Ratio of gene expression level in ectopic tissue to normal tissue
CtoU: Ratio of gene expression level in ectopic tissue to eutopic tissue
UtoN: Ratio of gene expression level in eutopic tissue to normal tissue
