# Supplementary material for: HOX cluster and their cofactors showed an altered expression pattern in eutopic and ectopic endometriosis tissues
Source: Reprod Biol Endocrinol. 2021 Sep 1;19:132. doi: 10.1186/s12958-021-00816-y (PMC8409001; doi:10.1186/s12958-021-00816-y)
Supplement: Supplementary file 1 — Additional file 1. [file 12958_2021_816_MOESM1_ESM.docx]

**Supplementary Data**

**Supplementary figures S1-S3. Gene expression array analysis for *HOX*-cofactor genes.** There were three biological repeats in each group. A, B and C in each graph show nine data that were analyzed with SPSS software. P<0.05 was considered to be significant.

**Supplementary figure S4.** Hierarchical cluster analysis of nine samples into three clusters. A) Based on DEGs. B) Based on non-DEGs.

**Table S1: Clinical characteristics of participating patients with and without endometriosis.**

| Confounding factors | | | Control group (n=15) | Endometriosis group (n=15) | |
| --- | --- | --- | --- | --- | --- |
| Age (year) | | | 22.5 ± 2.5 | 29.5 ± 1.1 | |
| BMI | | | 24.1 ± 0.5 | 24.7± 0.4 | |
| Number of children | | | At least one child | 0 | |
| Infertility Duration  (year) | | | 4.1 ± 0.5 | 6.0 ± 0.6 | |
| Prolactin | | | 15.9 ± 0.8 | 15.7 ± 0.9 | |
| LH | | | 5.4 ± 0.5 | 2.5 ± 0.2 | |
| FSH | | | 5.2 ± 0.5 | 4.6 ± 0.7 | |
| TSH | | | 2.6 ± 0.2 | 3.2 ± 0.3 | |
| Hypertension | | | Neg | Neg | |
| Diabetes mellitus | | | Neg | Neg | |
| Autoimmune diseases  (lupus, [Rheumatoid arthritis, Hashimoto's thyroiditis)](https://www.mayoclinic.org/diseases-conditions/rheumatoid-arthritis/symptoms-causes/syc-20353648) | | | Neg | Neg | |
| Asthma | | | Neg | Neg | |
| Familial history of endometriosis | | | Neg | Negative | N=13 |
|  |  |  |  | Positive | N=2 |
| Menstruation phase | Proliferative | | N=5 | N=5 | |
|  | Secretory | | N=10 | N=10 | |
| Obstetrical history | | | Neg | 3 of 15 patients (Abortion, Endometriosis) | |
| Previous surgery | | | 4 of 15 controls (Hysteroscopy, Appendectomy, Laparoscopy, Rhinoplasty) | 3 of 15 patients (D&C, Endometriosis) | |
| Dyspareunia | | | Neg | Positive | N=5 |
|  |  |  |  | Negative | N=10 |
| Dysmenorrhea | | Negative | N=9 | N=2 | |
|  |  | Mild | N=2 | N=0 | |
|  |  | Moderate | N=4 | N=6 | |
|  |  | Severe | N=0 | N=7 | |
| Hirsutism | | Negative | N=13 | N=12 | |
|  |  | Mild | N=2 | N=2 | |
|  |  | Moderate | N=0 | N=1 | |
| Chronic pelvic pain | | | Neg | Negative | N=11 |
|  |  |  |  | Positive | N=4 |
| Type of infertility | | | Secondary | Primary | N=13 |
|  |  |  |  | Secondary | N=1 |
| Hysterosalpingograms | | | Normal | Normal | N=12 |
|  |  |  |  | Unilateral retention | N=2 |
|  |  |  |  | Bilateral retention | N=1 |
| Smoking | | | No | No | |

The quantitative data are shown as mean ± S.E.M,

Abbreviations: Neg=Negative, D&C=dilation and curettage.

**Table S2: 52 DEGs and the rate of gene expression changes between normal and patient tissues.**

| **Eutopic to Control samples** | | | |  | | **Ectopic to Control samples** | | |  |  |
| --- | --- | --- | --- | --- | --- | --- | --- | --- | --- | --- |
| **Gene symbol** | **Fold Change** | | **Log fold change** |  | | **Gene symbol** | **Fold Change** | **Log fold change** |  |  |
| ***HOXB7*** | 0.102256 | | -3.28974 |  | | ***HOXB7*** | 0.052462 | -4.25258 |  |  |
| ***HOXB4*** | 0.320501 | | -1.6416 |  | | ***HOXB4*** | 0.01924 | -5.69977 |  |  |
| ***HOXB8*** | 0.397444 | | -1.33118 |  | | ***HOXB8*** | 0.008338 | -6.90612 |  |  |
| ***BARX1*** | 218.38 | | 7.770697 |  | | ***BARX1*** | 3474.67 | 11.76266 |  |  |
| ***ARX*** | 47.0884 | | 5.5573 |  | | ***ARX*** | 2124.948 | 11.05321 |  |  |
| ***PITX2*** | 60.10186 | | 5.909338 |  | | ***PITX2*** | 1388.929 | 10.43976 |  |  |
| ***SIX6*** | 305.6533 | | 8.255752 |  | | ***SIX6*** | 717.3853 | 9.486604 |  |  |
| ***CDX2*** | 272.2182 | | 8.08862 |  | | ***CDX2*** | 718.3046 | 9.488452 |  |  |
| ***EMX1*** | 2324.612 | | 11.18277 |  | | ***EMX1*** | 758.9458 | 9.567853 |  |  |
| ***TLX1*** | 3868.9 | | 11.91771 |  | | ***TLX1*** | 527.1588 | 9.042094 |  |  |
| ***PHOX2B*** | 1759.657 | | 10.78108 |  | | ***PHOX2B*** | 470.2195 | 8.877191 |  |  |
| ***DMBX1*** | 9207.146 | | 13.16854 |  | | ***DMBX1*** | 666.5631 | 9.380598 |  |  |
| ***HOXC6*** | 8.365557 | | 3.064462 |  | | ***HOXC6*** | 32.10712 | 5.004821 |  |  |
| ***LBX1*** | 142541.5 | | 17.12102 |  | | ***LBX1*** | 26.16059 | 4.709323 |  |  |
| ***VAX1*** | 15842.35 | | 13.9515 |  | | ***VAX1*** | 23.414 | 4.5493 |  |  |
| ***PITX3*** | 2658.53 | | 11.37641 |  | | ***PITX3*** | 23.16716 | 4.534009 |  |  |
| ***LMX1B*** | 216.2785 | | 7.756746 |  | | ***LMX1B*** | 19.86166 | 4.311914 |  |  |
| ***SHOX*** | 1018.75 | | 9.992585 |  | | ***SHOX*** | 21.2044 | 4.406292 |  |  |
| ***VAX2*** | 15.49991 | | 3.954188 |  | | ***VAX2*** | 14.68879 | 3.876643 |  |  |
| ***HOXC13*** | 57851.9 | | 15.82008 |  | | ***HOXC13*** | 12.09035 | 3.595784 |  |  |
| ***EN1*** | 40126.41 | | 15.29226 |  | | ***EN1*** | 189.6205 | 7.566971 |  |  |
| ***VSX1*** | 1538.416 | | 10.58723 |  | | ***VSX1*** | 9.778731 | 3.289647 |  |  |
| ***HLX*** | 9.787039 | | 3.290872 |  | | ***HLX*** | 7.003254 | 2.808025 |  |  |
| ***HOXC12*** | 76082.51 | | 16.21528 |  | | ***HOXC12*** | 141.7464 | 7.147169 |  |  |
| ***HOXD3*** | 2.863847 | | 1.517954 |  | | ***HOXD3*** | 5.118532 | 2.35573 |  |  |
| ***SIX3*** | 61.40422 | | 5.940266 |  | | ***SIX3*** | 19.7279 | 4.302166 |  |  |
| ***OTP*** | 40.79639 | | 5.35037 |  | | ***OTP*** | 2.263198 | 1.178363 |  |  |
| ***DLX1*** | 667.5541 | | 9.382741 |  | | ***DLX1*** | 20.85345 | 4.382214 |  |  |
| ***CUX1*** | 0.286672 | | -1.80253 |  | | ***HOXB2*** | 0.102646 | -3.28424 |  |  |
| ***ISL2*** | 71949.16 | | 16.13469 |  | | ***MSX1*** | 0.023292 | -5.42402 |  |  |
| ***PROP1*** | 51.3847 | | 5.683267 |  | | ***HOXB3*** | 0.026887 | -5.21693 |  |  |
| ***OTX1*** | 3.06 | | 1.613532 |  | | ***MSX2*** | 0.00492 | -7.66724 |  |  |
| ***PITX1*** | 2.77363 | | 1.471775 |  | | ***HOXA9*** | 0.138963 | -2.84723 |  |  |
| ***LMX1A*** | 216.2785 | | 7.756746 |  | | ***OTX2*** | 1502.242 | 10.5529 |  |  |
| ***LHX1*** | 4.18867 | | 2.066492 |  | | ***HOXC9*** | 599.0024 | 9.226418 |  |  |
| ***EN2*** | 6.304321 | | 2.656341 |  | | ***HOXC8*** | 172.8262 | 7.433178 |  |  |
|  |  | |  |  | | ***MKX*** | 121.3395 | 6.922906 |  |  |
|  |  | |  |  | | ***HOXD1*** | 40.94785 | 5.355716 |  |  |
|  |  | |  |  | | ***HOXC10*** | 23.29765 | 4.542113 |  |  |
|  |  | |  |  | | ***PAX3*** | 20.81021 | 4.37922 |  |  |
|  | |  | | | | ***HHEX*** | 9.771132 | 3.288526 |  |  |
|  | |  | | | | ***PROX1*** | 9.455249 | 3.241115 |  |  |
|  |  | |  | |  | ***HOPX*** | 5.985543 | 2.581482 |  |  |
|  |  | |  | |  | ***MIXL1*** | 0.052462 | -4.25258 |  |  |

**Table S3. Integration of annotation of the DEGs.**

| **Description** | **Count** | | **Percent** | | **P-value** |
| --- | --- | --- | --- | --- | --- |
| Homeobox | | 52 | | 100 | 1.29E-99 |
| DNA-binding | | 52 | | 100 | 2.20E-49 |
| Transcription | | 51 | | 98.08 | 1.08E-26 |
| Developmental protein | | 50 | | 96.15 | 4.18E-61 |
| Transcription factor | | 27 | | 51.92 | 2.00E-04 |
| Neurogenesis | | 27 | | 51.92 | 0.003395 |
| Differentiation | | 22 | | 42.31 | 0.005909 |
| Organ morphogenesis | | 21 | | 40.38 | 1.73E-04 |
| Embryonic development | | 19 | | 36.54 | 0.027011 |

**Table S4. Annotation of the DEGs and the genes in five networks with GO.**

| **Description** | **Count** | | | **Percent** | | | | **P-value** |  |
| --- | --- | --- | --- | --- | --- | --- | --- | --- | --- |
| **DEGs in ectopic** | | | | | | | | |  |
| GO:0043565~sequence-specific DNA binding | | | 34 | | | 75.56 | | 2.37E-41 |  |
| GO:0009952~anterior/posterior pattern specification | | | 16 | | | 35.56 | | 7.54E-25 |  |
| GO:0005634~nucleus | | | 42 | | | 93.33 | | 1.02E-18 |  |
| GO:0006351~transcription, DNA-templated | | | 28 | | | 62.22 | | 4.78E-15 |  |
| GO:0000122~negative regulation of transcription from RNA polymerase II promoter | | | 17 | | | 37.78 | | 1.50E-11 |  |
| GO:0048704~embryonic skeletal system morphogenesis | | | 7 | | | 15.56 | | 6.94E-10 |  |
| GO:0006366~transcription from RNA polymerase II promoter | | | 13 | | | 28.89 | | 5.04E-09 |  |
| GO:0007275~multicellular organism development | | | 13 | | | 28.89 | | 5.99E-09 |  |
| GO:0045944~positive regulation of transcription from RNA polymerase II promoter | | | 16 | | | 35.56 | | 1.32E-08 |  |
| GO:0071542~dopaminergic neuron differentiation | | | 5 | | | 11.11 | | 2.90E-07 |  |
| GO:0001227~transcriptional repressor activity, RNA polymerase II transcription regulatory region sequence-specific binding | | | 6 | | | 13.33 | | 4.29E-07 |  |
| GO:0001077~transcriptional activator activity, RNA polymerase II core promoter proximal region sequence-specific binding | | | 7 | | | 15.56 | | 3.17E-05 |  |
| GO:0009954~proximal/distal pattern formation | | | 4 | | | 8.89 | | 3.27E-05 |  |
| GO:0048706~embryonic skeletal system development | | | 4 | | | 8.89 | | 6.49E-05 |  |
| GO:0030901~midbrain development | | | 4 | | | 8.89 | | 7.17E-05 |  |
| GO:0035115~embryonic forelimb morphogenesis | | | 4 | | | 8.89 | | 7.90E-05 |  |
| GO:0000980~RNA polymerase II distal enhancer sequence-specific DNA binding | | | 4 | | | 8.89 | | 6.45E-04 |  |
| GO:0060216~definitive hemopoiesis | | | 3 | | | 6.67 | | 7.87E-04 |  |
| GO:0001701~in utero embryonic development | | | 5 | | | 11.11 | | 0.001428 |  |
| GO:0007517~muscle organ development | | | 4 | | | 8.89 | | 0.001629 |  |
| GO:0009887~organ morphogenesis | | | 4 | | | 8.89 | | 0.001792 |  |
| GO:0030878~thyroid gland development | | | 3 | | | 6.67 | | 0.001937 |  |
| GO:0030182~neuron differentiation | | | 4 | | | 8.89 | | 0.001964 |  |
| GO:0035116~embryonic hindlimb morphogenesis | | | 3 | | | 6.67 | | 0.002429 |  |
| GO:0009953~dorsal/ventral pattern formation | | | 3 | | | 6.67 | | 0.003166 |  |
| GO:0002088~lens development in camera-type eye | | | 3 | | | 6.67 | | 0.003365 |  |
| GO:0048536~spleen development | | | 3 | | | 6. 67 | | 0.003995 |  |
| GO:0090427~activation of meiosis | | | 2 | | | 4.44 | | 0.005234 |  |
| GO:2001055~positive regulation of mesenchymal cell apoptotic process | | | 2 | | | 4.44 | | 0.005234 |  |
| GO:0048666~neuron development | | | 3 | | | 6.67 | | 0.006455 |  |
| GO:0035880~embryonic nail plate morphogenesis | | | 2 | | | 4.44 | | 0.007841 |  |
| GO:0008344~adult locomotory behavior | | | 3 | | | 6.67 | | 0.008188 |  |
| GO:0042475~odontogenesis of dentin-containing tooth | | | 3 | | | 6.67 | | 0.009125 |  |
| GO:0045665~negative regulation of neuron differentiation | | | 3 | | | 6.67 | | 0.009447 |  |
| GO:0021615~glossopharyngeal nerve morphogenesis | | | 2 | | | 4.44 | | 0.010441 |  |
| GO:0007420~brain development | | | 4 | | | 8.89 | | 0.013439 |  |
| GO:0007601~visual perception | | | 4 | | | 8.89 | | 0.015612 |  |
| GO:0061312~BMP signaling pathway involved in heart development | | | 2 | | | 4.44 | | 0.015621 |  |
| GO:0021516~dorsal spinal cord development | | | 2 | | | 4.44 | | 0.018202 |  |
| GO:0055009~atrial cardiac muscle tissue morphogenesis | | | 2 | | | 4.44 | | 0.018202 |  |
| GO:0008285~negative regulation of cell proliferation | | | 5 | | | 11.11 | | 0.019687 |  |
| GO:0001162~RNA polymerase II intronic transcription regulatory region sequence-specific DNA binding | | | 2 | | | 4.44 | | 0.020667 |  |
| GO:0002011~morphogenesis of an epithelial sheet | | | 2 | | | 4.44 | | 0.020775 |  |
| GO:0009653~anatomical structure morphogenesis | | | 3 | | | 6.67 | | 0.024199 |  |
| GO:0008190~eukaryotic initiation factor 4E binding | | | 2 | | | 4.44 | | 0.025768 |  |
| GO:0070365~hepatocyte differentiation | | | 2 | | | 4.44 | | 0.025903 |  |
| GO:0090009~primitive streak formation | | | 2 | | | 4.44 | | 0.025903 |  |
| GO:0046622~positive regulation of organ growth | | | 2 | | | 4.44 | | 0.028457 |  |
| GO:0048484~enteric nervous system development | | | 2 | | | 4.44 | | 0.028457 |  |
| GO:0001764~neuron migration | | | 3 | | | 6.67 | | 0.030897 |  |
| GO:0021846~cell proliferation in forebrain | | | 2 | | | 4.44 | | 0.031004 |  |
| GO:0070306~lens fiber cell differentiation | | | 2 | | | 4.44 | | 0.033545 |  |
| GO:0001829~trophectodermal cell differentiation | | | 2 | | | 4.44 | | 0.033545 |  |
| GO:0003198~epithelial to mesenchymal transition involved in endocardial cushion formation | | | 2 | | | 4.44 | | 0.033545 |  |
| GO:2000678~negative regulation of transcription regulatory region DNA binding | | | 2 | | | 4.44 | | 0.03608 |  |
| GO:0045893~positive regulation of transcription, DNA-templated | | | 5 | | | 11.11 | | 0.045228 |  |
| GO:0071837~HMG box domain binding | | | 2 | | | 4.44 | | 0.045914 |  |
| GO:0048557~embryonic digestive tract morphogenesis | | | 2 | | | 4.44 | | 0.046152 |  |
| GO:0023019~signal transduction involved in regulation of gene expression | | | 2 | | | 4.44 | | 0.048654 |  |
| GO:0045638~negative regulation of myeloid cell differentiation | | | 2 | | | 4.44 | | 0.048654 |  |
| GO:0001501~skeletal system development | | | 3 | | | 6.67 | | 0.050029 |  |
| GO:0007492~endoderm development | | | 2 | | | 4.44 | | 0.053639 |  |
| GO:0031016~pancreas development | | | 2 | | | 4.44 | | 0.053639 |  |
| GO:0007411~axon guidance | | | 3 | | | 6.67 | | 0.065084 |  |
| GO:0048863~stem cell differentiation | | | 2 | | | 4.44 | | 0.068441 |  |
| GO:0021983~pituitary gland development | | | 2 | | | 4.44 | | 0.070886 |  |
| GO:0060412~ventricular septum morphogenesis | | | 2 | | | 4.44 | | 0.073325 |  |
| GO:0001654~eye development | | | 2 | | | 4.44 | | 0.075757 |  |
| GO:0034504~protein localization to nucleus | | | 2 | | | 4.44 | | 0.075757 |  |
| GO:0030513~positive regulation of BMP signaling pathway | | | 2 | | | 4.44 | | 0.078183 |  |
| GO:0042127~regulation of cell proliferation | | | 3 | | | 6.67 | | 0.08455 |  |
| GO:0016055~Wnt signaling pathway | | | 3 | | | 6.67 | | 0.086115 |  |
| GO:0043010~camera-type eye development | | | 2 | | | 4.44 | | 0.09974 |  |
| **DEGs in eutopic** | | | | | | | | |  |
| GO:0043565~sequence-specific DNA binding | | | 26 | 72.22 | | | | 1.15E-30 |  |
| GO:0005634~nucleus | | | 35 | 97.22 | | | | 2.76E-17 |  |
| GO:0003700~transcription factor activity, sequence-specific DNA binding | | | 18 | 50 | | | | 1.03E-12 |  |
| GO:0009952~anterior/posterior pattern specification | | | 9 | 25 | | | | 3.93E-12 |  |
| GO:0006355~regulation of transcription, DNA-templated | | | 19 | 52.78 | | | | 1.29E-10 |  |
| GO:0006366~transcription from RNA polymerase II promoter | | | 13 | 36.11 | | | | 2.56E-10 |  |
| GO:0045944~positive regulation of transcription from RNA polymerase II promoter | | | 15 | 41.67 | | | | 3.60E-09 |  |
| GO:0003677~DNA binding | | | 18 | 50 | | | | 6.36E-09 |  |
| GO:0007275~multicellular organism development | | | 11 | 30.56 | | | | 6.93E-08 |  |
| GO:0071542~dopaminergic neuron differentiation | | | 5 | 13.89 | | | | 1.13E-07 |  |
| GO:0006351~transcription, DNA-templated | | | 17 | 47.22 | | | | 4.95E-07 |  |
| GO:0000122~negative regulation of transcription from RNA polymerase II promoter | | | 11 | 30.56 | | | | 1.37E-06 |  |
| GO:0048666~neuron development | | | 5 | 13.89 | | | | 2.42E-06 |  |
| GO:0000977~RNA polymerase II regulatory region sequence-specific DNA binding | | | 7 | 19.44 | | | | 3.92E-06 |  |
| GO:0045665~negative regulation of neuron differentiation | | | 5 | 13.89 | | | | 5.38E-06 |  |
| GO:0001077~transcriptional activator activity, RNA polymerase II core promoter proximal region sequence-specific binding | | | 7 | 19.44 | | | | 8.10E-06 |  |
| GO:0030901~midbrain development | | | 4 | 11.11 | | | | 3.58E-05 |  |
| GO:0009653~anatomical structure morphogenesis | | | 5 | 13.89 | | | | 3.88E-05 |  |
| GO:0009953~dorsal/ventral pattern formation | | | 4 | 11.11 | | | | 3.95E-05 |  |
| GO:0048704~embryonic skeletal system morphogenesis | | | 4 | 11.11 | | | | 7.20E-05 |  |
| GO:0000978~RNA polymerase II core promoter proximal region sequence-specific DNA binding | | | 7 | 19.44 | | | | 8.04E-05 |  |
| GO:0001227~transcriptional repressor activity, RNA polymerase II transcription regulatory region sequence-specific binding | | | 4 | 11.11 | | | | 2.45E-04 |  |
| GO:0000980~RNA polymerase II distal enhancer sequence-specific DNA binding | | | 4 | 11.11 | | | | 3.27E-04 |  |
| GO:0005667~transcription factor complex | | | 5 | 13.89 | | | | 4.94E-04 |  |
| GO:0009887~organ morphogenesis | | | 4 | 11.11 | | | | 9.17E-04 |  |
| GO:0001205~transcriptional activator activity, RNA polymerase II distal enhancer sequence-specific binding | | | 3 | 8.33 | | | | 0.001216 |  |
| GO:0048536~spleen development | | | 3 | 8.33 | | | | 0.002543 |  |
| GO:0021549~cerebellum development | | | 3 | 8.33 | | | | 0.002685 |  |
| GO:0008344~adult locomotory behavior | | | 3 | 8.33 | | | | 0.005242 |  |
| GO:0060126~somatotropin secreting cell differentiation | | | 2 | 5.56 | | | | 0.00624 |  |
| GO:0031490~chromatin DNA binding | | | 3 | 8.33 | | | | 0.006419 |  |
| GO:0021979~hypothalamus cell differentiation | | | 2 | 5.56 | | | | 0.008312 |  |
| GO:0007601~visual perception | | | 4 | 11.11 | | | | 0.008344 |  |
| GO:0003682~chromatin binding | | | 5 | 13.89 | | | | 0.008418 |  |
| GO:0001162~RNA polymerase II intronic transcription regulatory region sequence-specific DNA binding | | | 2 | 5.56 | | | | 0.01647 |  |
| GO:1990403~embryonic brain development | | | 2 | 5.56 | | | | 0.016557 |  |
| GO:0001206~transcriptional repressor activity, RNA polymerase II distal enhancer sequence-specific binding | | | 2 | 5.56 | | | | 0.01851 |  |
| GO:0001764~neuron migration | | | 3 | 8.33 | | | | 0.02015 |  |
| GO:0046622~positive regulation of organ growth | | | 2 | 5.56 | | | | 0.022697 |  |
| GO:0048484~enteric nervous system development | | | 2 | 5.56 | | | | 0.022697 |  |
| GO:0021846~cell proliferation in forebrain | | | 2 | 5.56 | | | | 0.024735 |  |
| GO:0070306~lens fiber cell differentiation | | | 2 | 5.56 | | | | 0.026769 |  |
| GO:0043524~negative regulation of neuron apoptotic process | | | 3 | 8.33 | | | | 0.030814 |  |
| GO:0001501~skeletal system development | | | 3 | 8.33 | | | | 0.032988 |  |
| GO:0021537~telencephalon development | | | 2 | 5.56 | | | | 0.034865 |  |
| GO:0048557~embryonic digestive tract morphogenesis | | | 2 | 5.56 | | | | 0.036879 |  |
| GO:0090190~positive regulation of branching involved in ureteric bud morphogenesis | | | 2 | 5.56 | | | | 0.038888 |  |
| GO:0007411~axon guidance | | | 3 | 8.33 | | | | 0.043237 |  |
| GO:0000981~RNA polymerase II transcription factor activity, sequence-specific DNA binding | | | 3 | 8.33 | | | | 0.048804 |  |
| GO:0009954~proximal/distal pattern formation | | | 2 | 5.56 | | | | 0.048876 |  |
| GO:0043565~sequence-specific DNA binding | | | 26 | 72.22 | | | | 1.15E-30 |  |
| **Genes in network A** | | | | | | | | |  |
| GO:0005634~nucleus | | | 26 | 92.86 | | | | 1.15E-11 |  |
| GO:0043565~sequence-specific DNA binding | | | 19 | 67.86 | | | | 1.56E-21 |  |
| GO:0006355~regulation of transcription, DNA-templated | | | 16 | 57.14 | | | | 1.10E-09 |  |
| GO:0003677~DNA binding | | | 16 | 57.14 | | | | 4.55E-09 |  |
| GO:0045944~positive regulation of transcription from RNA polymerase II promoter | | | 15 | 53.57 | | | | 4.83E-11 |  |
| GO:0006366~transcription from RNA polymerase II promoter | | | 12 | 42.86 | | | | 1.63E-10 |  |
| GO:0003700~transcription factor activity, sequence-specific DNA binding | | | 10 | 35.71 | | | | 1.12E-05 |  |
| GO:0001077~transcriptional activator activity, RNA polymerase II core promoter proximal region sequence-specific binding | | | 8 | 28.57 | | | | 6.68E-08 |  |
| GO:0000978~RNA polymerase II core promoter proximal region sequence-specific DNA binding | | | 8 | 28.57 | | | | 1.06E-06 |  |
| GO:0045665~negative regulation of neuron differentiation | | | 6 | 21.43 | | | | 2.62E-08 |  |
| GO:0001501~skeletal system development | | | 6 | 21.43 | | | | 2.35E-06 |  |
| GO:0005667~transcription factor complex | | | 6 | 21.43 | | | | 8.45E-06 |  |
| GO:0000977~RNA polymerase II regulatory region sequence-specific DNA binding | | | 6 | 21.43 | | | | 1.75E-05 |  |
| GO:0003682~chromatin binding | | | 6 | 21.43 | | | | 3.44E-04 |  |
| GO:0007275~multicellular organism development | | | 6 | 21.43 | | | | 0.001293 |  |
| GO:0000122~negative regulation of transcription from RNA polymerase II promoter | | | 6 | 21.43 | | | | 0.00526 |  |
| GO:0071542~dopaminergic neuron differentiation | | | 5 | 17.86 | | | | 3.80E-08 |  |
| GO:0048666~neuron development | | | 5 | 17.86 | | | | 8.26E-07 |  |
| GO:0030901~midbrain development | | | 4 | 14.29 | | | | 1.62E-05 |  |
| GO:0009952~anterior/posterior pattern specification | | | 4 | 14.29 | | | | 2.80E-04 |  |
| GO:0009653~anatomical structure morphogenesis | | | 4 | 14.29 | | | | 4.23E-04 |  |
| GO:0043524~negative regulation of neuron apoptotic process | | | 4 | 14.29 | | | | 0.00121 |  |
| GO:0021983~pituitary gland development | | | 3 | 10.71 | | | | 9.17E-04 |  |
| GO:0009953~dorsal/ventral pattern formation | | | 3 | 10.71 | | | | 0.001199 |  |
| GO:0042733~embryonic digit morphogenesis | | | 3 | 10.71 | | | | 0.003634 |  |
| GO:0007517~muscle organ development | | | 3 | 10.71 | | | | 0.008946 |  |
| GO:0007420~brain development | | | 3 | 10.71 | | | | 0.037143 |  |
| GO:0021524~visceral motor neuron differentiation | | | 2 | 7.14 | | | | 0.004816 |  |
| GO:0021979~hypothalamus cell differentiation | | | 2 | 7.14 | | | | 0.006417 |  |
| GO:0021520~spinal cord motor neuron cell fate specification | | | 2 | 7.14 | | | | 0.011203 |  |
| GO:1990403~embryonic brain development | | | 2 | 7.14 | | | | 0.012794 |  |
| GO:0070306~lens fiber cell differentiation | | | 2 | 7.14 | | | | 0.02071 |  |
| GO:0021522~spinal cord motor neuron differentiation | | | 2 | 7.14 | | | | 0.026998 |  |
| GO:0071837~HMG box domain binding | | | 2 | 7.14 | | | | 0.028416 |  |
| GO:0031290~retinal ganglion cell axon guidance | | | 2 | 7.14 | | | | 0.030128 |  |
| GO:0009954~proximal/distal pattern formation | | | 2 | 7.14 | | | | 0.03791 |  |
| GO:0001205~transcriptional activator activity, RNA polymerase II distal enhancer sequence-specific binding | | | 2 | 7.14 | | | | 0.039255 |  |
| GO:0035116~embryonic hindlimb morphogenesis | | | 2 | 7.14 | | | | 0.044092 |  |
| **Genes in network B** | | | | | | | | |  |
| GO:0043565~sequence-specific DNA binding | | 19 | | | 67.86 | | 1.56E-21 | | |
| GO:0005634~nucleus | | 26 | | | 92.86 | | 1.15E-11 | | |
| GO:0045944~positive regulation of transcription from RNA polymerase II promoter | | 15 | | | 53.57 | | 4.83E-11 | | |
| GO:0006366~transcription from RNA polymerase II promoter | | 12 | | | 42.86 | | 1.63E-10 | | |
| GO:0006355~regulation of transcription, DNA-templated | | 16 | | | 57.14 | | 1.10E-09 | | |
| GO:0003677~DNA binding | | 16 | | | 57.14 | | 4.55E-09 | | |
| GO:0045665~negative regulation of neuron differentiation | | 6 | | | 21.43 | | 2.62E-08 | | |
| GO:0071542~dopaminergic neuron differentiation | | 5 | | | 17.86 | | 3.80E-08 | | |
| GO:0001077~transcriptional activator activity, RNA polymerase II core promoter proximal region sequence-specific binding | | 8 | | | 28.57 | | 6.68E-08 | | |
| GO:0048666~neuron development | | 5 | | | 17.86 | | 8.26E-07 | | |
| GO:0000978~RNA polymerase II core promoter proximal region sequence-specific DNA binding | | 8 | | | 28.57 | | 1.06E-06 | | |
| GO:0001501~skeletal system development | | 6 | | | 21.43 | | 2.35E-06 | | |
| GO:0005667~transcription factor complex | | 6 | | | 21.43 | | 8.45E-06 | | |
| GO:0003700~transcription factor activity, sequence-specific DNA binding | | 10 | | | 35.71 | | 1.12E-05 | | |
| GO:0030901~midbrain development | | 4 | | | 14.29 | | 1.62E-05 | | |
| GO:0000977~RNA polymerase II regulatory region sequence-specific DNA binding | | 6 | | | 21.43 | | 1.75E-05 | | |
| GO:0009952~anterior/posterior pattern specification | | 4 | | | 14.29 | | 2.80E-04 | | |
| GO:0003682~chromatin binding | | 6 | | | 21.43 | | 3.44E-04 | | |
| GO:0009653~anatomical structure morphogenesis | | 4 | | | 14.29 | | 4.23E-04 | | |
| GO:0006351~transcription, DNA-templated | | 11 | | | 39.29 | | 5.79E-04 | | |
| GO:0021983~pituitary gland development | | 3 | | | 10.71 | | 9.17E-04 | | |
| GO:0009953~dorsal/ventral pattern formation | | 3 | | | 10.71 | | 0.001199 | | |
| GO:0043524~negative regulation of neuron apoptotic process | | 4 | | | 14.29 | | 0.00121 | | |
| GO:0007275~multicellular organism development | | 6 | | | 21.43 | | 0.001293 | | |
| GO:0042733~embryonic digit morphogenesis | | 3 | | | 10.71 | | 0.003634 | | |
| GO:0021524~visceral motor neuron differentiation | | 2 | | | 7.14 | | 0.004816 | | |
| GO:0000122~negative regulation of transcription from RNA polymerase II promoter | | 6 | | | 21.43 | | 0.00526 | | |
| GO:0021979~hypothalamus cell differentiation | | 2 | | | 7.14 | | 0.006417 | | |
| GO:0007517~muscle organ development | | 3 | | | 10.71 | | 0.008946 | | |
| GO:0021520~spinal cord motor neuron cell fate specification | | 2 | | | 7.14 | | 0.011203 | | |
| GO:1990403~embryonic brain development | | 2 | | | 7.14 | | 0.012794 | | |
| GO:0070306~lens fiber cell differentiation | | 2 | | | 7.14 | | 0.02071 | | |
| GO:0021522~spinal cord motor neuron differentiation | | 2 | | | 7.14 | | 0.026998 | | |
| GO:0071837~HMG box domain binding | | 2 | | | 7.14 | | 0.028416 | | |
| GO:0031290~retinal ganglion cell axon guidance | | 2 | | | 7.14 | | 0.030128 | | |
| GO:0007420~brain development | | 3 | | | 10.71 | | 0.037143 | | |
| GO:0009954~proximal/distal pattern formation | | 2 | | | 7.14 | | 0.03791 | | |
| GO:0001205~transcriptional activator activity, RNA polymerase II distal enhancer sequence-specific binding | | 2 | | | 7.14 | | 0.039255 | | |
| GO:0035116~embryonic hindlimb morphogenesis | | 2 | | | 7.14 | | 0.044092 | | |
| **Genes in network C** | | | | | | | | |  |
| GO:0043565~sequence-specific DNA binding | 13 | | | 86.67 | | | | 5.27E-17 |  |
| GO:0009952~anterior/posterior pattern specification | 8 | | | 53.33 | | | | 1.42E-13 |  |
| GO:0006351~transcription, DNA-templated | 11 | | | 73.33 | | | | 2.88E-07 |  |
| GO:0005634~nucleus | 14 | | | 93.33 | | | | 1.41E-06 |  |
| GO:0048704~embryonic skeletal system morphogenesis | 4 | | | 26.67 | | | | 4.14E-06 |  |
| GO:0003700~transcription factor activity, sequence-specific DNA binding | 7 | | | 46.67 | | | | 6.78E-05 |  |
| GO:0045944~positive regulation of transcription from RNA polymerase II promoter | 7 | | | 46.67 | | | | 7.83E-05 |  |
| GO:0006355~regulation of transcription, DNA-templated | 8 | | | 53.33 | | | | 8.89E-05 |  |
| GO:0000122~negative regulation of transcription from RNA polymerase II promoter | 6 | | | 40 | | | | 2.07E-04 |  |
| GO:0007275~multicellular organism development | 5 | | | 33.33 | | | | 7.16E-04 |  |
| GO:0030182~neuron differentiation | 3 | | | 20 | | | | 0.002757 |  |
| GO:0045892~negative regulation of transcription, DNA-templated | 4 | | | 26.67 | | | | 0.007436 |  |
| GO:0070365~hepatocyte differentiation | 2 | | | 13.33 | | | | 0.008308 |  |
| GO:0003714~transcription corepressor activity | 3 | | | 20 | | | | 0.011907 |  |
| GO:0044212~transcription regulatory region DNA binding | 3 | | | 20 | | | | 0.01305 |  |
| GO:0007492~endoderm development | 2 | | | 13.33 | | | | 0.017373 |  |
| GO:0031016~pancreas development | 2 | | | 13.33 | | | | 0.017373 |  |
| GO:0010628~positive regulation of gene expression | 3 | | | 20 | | | | 0.019503 |  |
| GO:0030878~thyroid gland development | 2 | | | 13.33 | | | | 0.020651 |  |
| GO:0048706~embryonic skeletal system development | 2 | | | 13.33 | | | | 0.024733 |  |
| GO:0048705~skeletal system morphogenesis | 2 | | | 13.33 | | | | 0.026361 |  |
| GO:0030326~embryonic limb morphogenesis | 2 | | | 13.33 | | | | 0.03285 |  |
| GO:0003677~DNA binding | 5 | | | 33.33 | | | | 0.042906 |  |
